# New Advances in Monitoring Cardiac Output in Circulatory Mechanical Assistance Devices. A Validation Study in a Porcine Model

**DOI:** 10.3389/fphys.2021.634779

**Published:** 2021-03-04

**Authors:** Begoña Quintana-Villamandos, Mónica Barranco, Ignacio Fernández, Manuel Ruiz, Juan Francisco del Cañizo

**Affiliations:** ^1^Department of Anesthesiology and Intensive Care, Gregorio Marañón Hospital, Madrid, Spain; ^2^Department of Pharmacology and Toxicology, Faculty of Medicine, Universidad Complutense, Madrid, Spain; ^3^Department of Cardiovascular Surgery, Gregorio Marañón Hospital, Madrid, Spain; ^4^Department of Surgery, Faculty of Medicine, Universidad Complutense, Madrid, Spain

**Keywords:** cardiac output, continuous-flow, continuous pulmonary artery thermodilution, minipig, left ventricular assist devices

## Abstract

Cardiac output (CO) measurement by continuous pulmonary artery thermodilution (CO_*CTD*_) has been studied in patients with pulsatile-flow LVADs (left ventricular assist devices), confirming the clinical utility. However, it has not been validated in patients with continuous-flow LVADs. Therefore, the aim of this study was to assess the validity of CO_*CTD*_ in continuous-flow LVADs. Continuous-flow LVADs were implanted in six miniature pigs for partial assistance of the left ventricle. Both methods of measuring CO—measurement by CO_*CTD*_ and intermittent pulmonary artery thermodilution, standard technique (CO_*ITD*_)—were used in four consecutive moments of the study: before starting the LVAD (basal moment), and with the LVAD started in normovolemia, hypervolemia (fluid overloading), and hypovolemia (shock hemorrhage). At the basal moment, CO_*CTD*_ and CO_*ITD*_ were closely correlated (*r*^2^ = 0.97), with a mean bias of −0.13 ± 0.16 L/min and percentage error of 11%. After 15 min of partial support LVAD, CO_*CTD*_ and CO_*ITD*_ were closely correlated (*r*^2^ = 0.91), with a mean bias of 0.31 ± 0.35 L/min and percentage error of 20%. After inducing hypervolemia, CO_*CTD*_ and CO_*ITD*_ were closely correlated (*r*^2^ = 0.99), with a mean bias of 0.04 ± 0.07 L/min and percentage error of 5%. After inducing hypovolemia, CO_*CTD*_ and CO_*ITD*_ were closely correlated (*r*^2^ = 0.74), with a mean bias of 0.08 ± 0.22 L/min and percentage error of 19%. This study shows that continuous pulmonary thermodilution could be an alternative method of monitoring CO in a porcine model with a continuous-flow LVAD.

## Introduction

Left ventricular assist devices (LVADs) are increasingly used for patients with advanced heart failure and have been implanted in more than 16.000 patients worldwide (Goldstein et al., 2019). The use of LVADs has significantly improved survival and reduced morbidity in these patients (Kiamanesh et al., 2019). LVADs may function as a bridge to ventricular recovery, a heart transplant, or destination therapy by restoring and maintaining systemic perfusion (Loor and Gonzalez-Stawinski, 2012). The first generation of implantable LVADs was a pulsatile device with a cardiac output (CO) defined by the pulsatility of the LVAD. However, in recent years, continuous-flow LVADs have replaced pulsatile-flow LVADs due to their smaller size, longer durability, higher energy efficiency, less thrombogenicity, and less surgical trauma (Loor and Gonzalez-Stawinski, 2012). Centrifugal continuous-flow LVADs (HeartWare HVAD and HeartMate 3) and axial continuous-flow LVAD (HeartMate 2) are the preferred, however, HeartMate 3 LVAD shows lower rates of ischemia strokes, hemorrhagic strokes and thrombotic events (Itzhaki Ben Zadok et al., 2019) and it is associated with higher survival (Austin et al., 2020) with respect to HeartMate 2 and HeartWare HVAD.

Information about CO is essential for optimal treatment of patients requiring LVADs (Birati and Rame, 2014; Uriel et al., 2016). Intermittent bolus pulmonary artery thermodilution (CO_*ITD*_) is the standard technique for monitoring CO in critically ill patients (Boldt et al., 1994; Haller et al., 1995). Continuous pulmonary artery thermodilution (CO_*CTD*_) has been compared and validated with the gold standard of bolus dose thermodilution using the pulmonary artery catheter (PAC) (Yelderman et al., 1992; Boldt et al., 1994; Lefrant et al., 1995). The CO_*CTD*_ technique is a modification of the PAC: it has a thermal filament in the catheter that is intermittently heated, the temperature changes are captured by a thermistor near the tip of the catheter, and the CO is determined from a modified version of the Stuart–Hamilton equation (Argueta and Paniagua, 2019). The CO_*CTD*_ measurement by PAC has been studied in patients with pulsatile-flow LVADs, confirming the clinical utility (despite a 500 ml bias for the CO) (Mets et al., 2002). So far, however, this new system has not been validated in patients with a continuous-flow LVAD.

The aim was to assess the validity of CO_*CTD*_ for the determination of CO in a porcine model with a continuous-flow LVAD with normovolemia, hypervolemia (fluid overload), and hypovolemia (bleeding). It is necessary to investigate the applicability of marketed systems (CO_*CTD*_ measurement by PAC) in a large animal model in order to search potential indications, to establish improvements (in continuous-flow LVADs) and then to optimize the application in the patient.

## Materials and Methods

### Experimental Setting

The study was conducted with six healthy mini-pigs. The pigs were moved from the farm of the Technological Institute of Agrarian Development (EX 013-C) (Community of Madrid, Spain) to the Experimental Medicine and Surgery Unit, Gregorio Marañón University General Hospital (ES280790000-087). The study was performed following European Union guidelines on the protection of animals used for experimental and other scientific purposes (Directive 2010/63/EU and Spanish Royal Decree RD 53/2013 BOE) and was approved by the ethics committee of Gregorio Marañón University General Hospital, Madrid, Spain.

### Anesthesia Protocol

The previously described anesthetic protocol was applied (Morillas-Sendín et al., 2015). The animals were premedicated with intramuscular ketamine, 20 mg/kg (Ketolar, Parke-Davis, Madrid Spain), and atropine, 0.04 mg/kg (Atropina Braun, Serra Pamies, Reus, Spain). The pigs were provided with 100% oxygen via a facemask, a 20 G cannula was inserted into an ear vein, and anesthesia was induced with intravenous fentanyl, 2.5 μg/kg (Fentanest, Kern Pharma, Barcelona, Spain) and propofol, 4 mg/kg (Diprivan 1%, AstraZeneca, Madrid, Spain). After intubation, each animal was connected to a volume-controlled ventilator (Dräger SA1, Dräger Medical AG, Lübeck, Germany) with an FIO2 of 1, an inspiratory-to-expiratory ratio of 1:2, a tidal volume of 12–15 mL/kg, and a respiratory rate adjusted to maintain normocapnia. Anesthesia was maintained for all animals with intravenous fentanyl (2.5 μg/kg/30 min) and propofol in continuous infusion (11–12 mg/kg/h). All animals received an infusion of saline solution (8 mL/kg/h). A 9 F arterial catheter was inserted into the right femoral artery, and a PAC (7.5 F Swan-Ganz CCOmbo catheter, Edwards Lifesciences, Irvine, CA, United States) was inserted into the right internal jugular vein for thermodilution measurements. The PAC was connected to an oximetry monitor (Vigilance, Edwards Critical Care Division, Irvine, CA, United States). Epicardial echocardiography was performed using the Vivid S5 system (GE Healthcare, Germany) equipped with a 4-MHz probe (3Sc-RS, GE).

### Surgical Protocol

The previously described surgical protocol was applied (Morillas-Sendín et al., 2015). A Biomedicus 540 centrifugal pump was implanted in the mini-pigs undergoing continuous-flow support. After median sternotomy, the animal was heparinized (4 mg/kg), a partial aortic cross-clamp was applied (just for anastomosing the output cannula of the LVAD to the aorta), and a 2 cm aortotomy was performed. The output cannula of the LVAD was anastomosed to the ascending aorta, and the input cannula (23 F Medtronic Ultraflex, Metdtronic Inc., Minneapolis, MN, United States) was placed through the apex of the left ventricle. The implant of the input cannula is practiced by placing two circular sutures, and then the cannula was placed with two turnstiles around the cannula. Finally, both cannulas were connected to the device. Input flow was measured using an ultrasound transducer (EMTEC, Germany) attached to the input cannula of the device.

### Procedure

**FIGURE 1:**
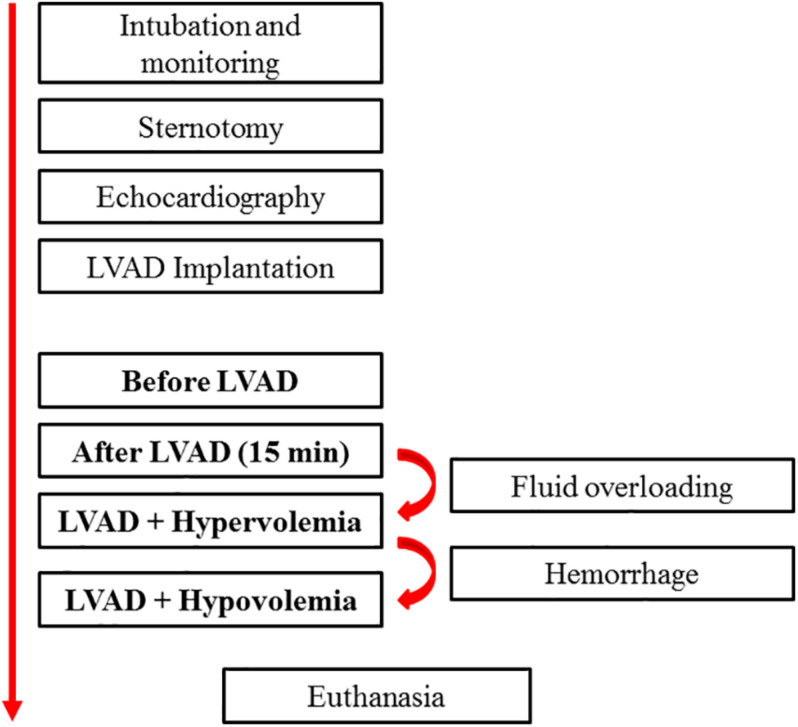
Flow chart of the experimental protocol. LVAD, left ventricular assist devices.

The experimental protocol is summarized in Figure 1. After sternotomy, an echocardiography study was performed, and pigs with aortic insufficiency, tricuspid regurgitation, atrial septal defects, or ventricular septal defects were excluded from the study. After implanting the LVAD, console parameters were adjusted to obtain a pump flow of 50% (partial support) of the baseline CO (cardiac output before LVAD is initiated) using the PAC. Measurements of CO using both methods—CO_*ITD*_ (standard technique) and CO_*CTD*_—were obtained in four consecutive moments of the study: before starting the LVAD, after 15 min of partial support LVAD, after inducing hypervolemia (fluid overloading, 75% serum saline + 25% gelatin to increase mean arterial pressure to 130 mmHg and/or central venous up to 20 mmHg) (partial support LVAD), and after inducing hypovolemia (controlled hemorrhage to decrease mean arterial pressure to around 50 mmHg) (partial support LVAD) (Bendjelid et al., 2010).

### Statistical Analysis

The results are expressed as mean + standard deviation (SD). CO determinations using the two methods, CO_*ITD*_ (standard technique) and CO_*CTD*_, were compared at different moments using paired Student’s *t*-test. Statistical significance was set at a *p*-value of < 0.05. Bias was defined as the mean difference between the two methods. A comparison of the bias between the two methods was assessed by Bland–Altman analysis (Odor et al., 2017). The percentage error was calculated as two SDs of the bias divided by the mean of CO obtained by the standard technique. A percentage error > ±30% is recommended as clinically acceptable when comparing a new method to the current reference method (Odor et al., 2017). Linear regression analysis was used to assess the relationship between CO obtained by the two methods. The statistical analysis was performed using IBM SPSS Statistics for Windows, version 20.0 (IBM Corp., Armonk, NY, United States) and GraphPad Prism 6.0 (GraphPad Software, California, United States).

## Results

A total of 72 paired measurements (three measurements from each stage per animal) were obtained from six male mini-pigs weighing 42.2 + 8.1 Kg. At each stage, the values of CO_*ITD*_ and CO_*CTD*_ were comparable (Table 1).

**TABLE 1:** Cardiac output over the study period.

	**Before LVAD**	**LVAD 15 min**	**LVAD hypervolemia**	**LVAD hypovolemia**
CO_*ITD*_ (L/min)	3 ± 1.01	3.44 ± 1.07	5.55 ± 1.55*	2.28 ± 0.44*
CO_*CTD*_ (L/min)	3.14 ± 1.05	3.12 ± 1.20	5.50 ± 1.53*	2.19 ± 0.42*

*LVAD, left ventricle assist device; CO_*ITD*_, cardiac output by intermittent bolus pulmonary artery thermodilution; CO_*CTD*_, cardiac output by continuous pulmonary artery thermodilution. Results are expressed as the mean* ± standard deviation. *p < 0.05 LVAD (15 min) vs. before LVAD or hypervolemia LVAD vs. LVAD (15 min) vs. LVAD hypovolemia vs. LVAD hypervolemia. *n* = *6 mini-pigs.*

**FIGURE 2:**
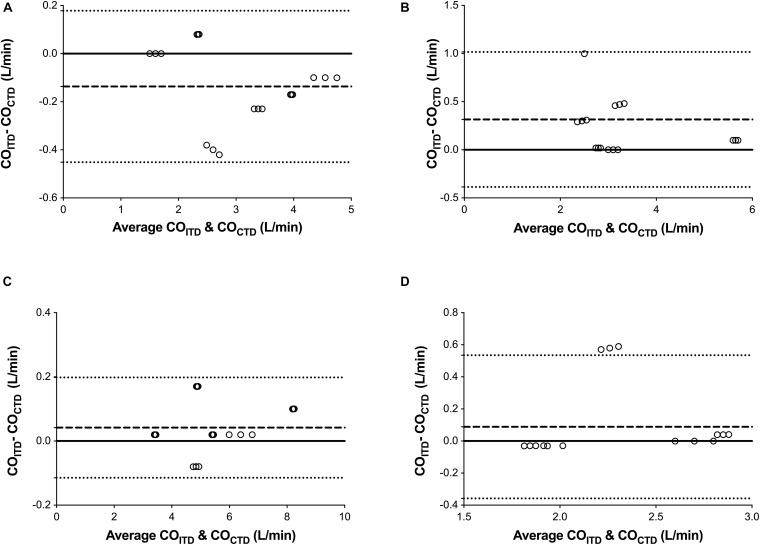
Bland-Altman representation depicting the agreement between the cardiac output (CO) measurement by continuous pulmonary artery thermodilution (CO_*CTD*_) and intermittent pulmonary artery thermodilution (CO_*ITD*_) in four consecutive moments of the study: before starting the LVAD **(A)**, with the LVAD started in normovolemia **(B)**, hypervolemia (fluid overloading) **(C)**, and hypovolemia (shock hemorrhage) **(D)**.

**FIGURE 3:**
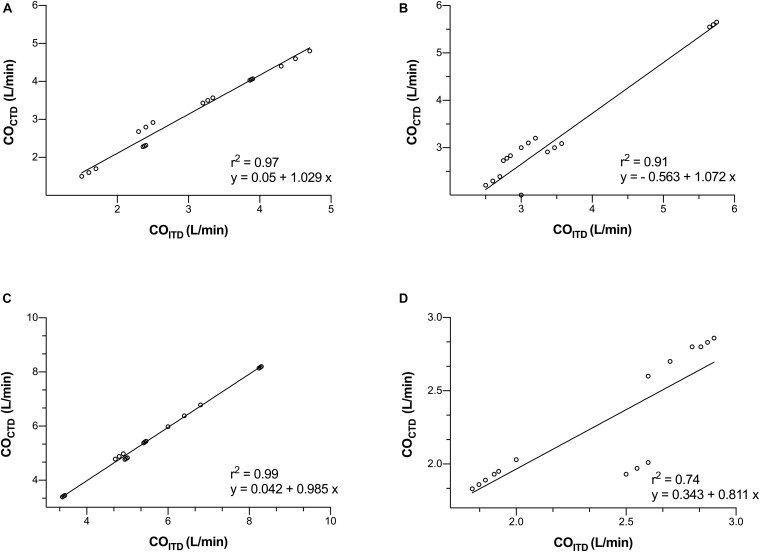
Correlation between the cardiac output (CO) measurement by continuous pulmonary artery thermodilution (CO_*CTD*_) and intermittent pulmonary artery thermodilution (CO_*ITD*_) in four consecutive moments of the study: before starting the LVAD **(A)**, with the LVAD started in normovolemia **(B)**, hypervolemia (fluid overloading) **(C)**, and hypovolemia (shock hemorrhage) **(D)**.

Before starting the LVAD, there were no significant differences between CO_*CTD*_ vs. CO_*ITD*_ (3.14 ± 1.05 vs. 3 ± 1.01 L/min, respectively, *p* = 0.69). CO_*CTD*_ and CO_*ITD*_ were closely correlated (*r*^2^ = 0.97), with a mean bias (±SD) of −0.13 ± 0.16 L/min and a percentage error of 11% (Figures 2A, 3A).

After 15 min of partial support, there were no significant differences between CO_*CTD*_ vs. CO_*ITD*_ (3.12 ± 1.20 vs. 3.44 ± 1.07 L/min, respectively, *p* = 0.41). CO_*CTD*_ and CO_*ITD*_ were closely correlated (*r*^2^ = 0.91), with a mean bias (±SD) of 0.31 ± 0.35 L/min and a percentage error of 20% (Figures 2B, 3B).

Hypervolemia was achieved by the average infusión of 3L (75% serum saline + 25% gelatin). According to the protocol, we induce hypervolemia in order to increase central venous pressure up to 20 mmHg. Pulmonary capillary wedge pressure was 21 ± 6 mmHg. After inducing hypervolemia, there were no significant differences between CO_*CTD*_ vs. CO_*ITD*_ (5.50 ± 1.53 vs. 5.55 ± 1.55 L/min respectively, *p* = 0.93). CO_*CTD*_ and CO_*ITD*_ were closely correlated (*r*^2^ = 0.99), with a mean bias (±SD) of 0.04 ± 0.07 L/min and a percentage error of 5% (Figures 2C, 3C).

Hypovolemia was achieved by an average 1L controlled hemorrhage. According to the protocol, we induce hipovolemia, controlled hemorrhage to decrease mean arterial pressure around 50 mmHg. Central venous pressure was 7 ± 2 mmHg, and pulmonary capillary wedge pressure was 9 ± 3 mmHg. After inducing hypovolemia, there were no significant differences between CO_*CTD*_ vs. CO_*ITD*_ (2.19 ± 0.42 vs. 2.28 ± 0.44 L/min respectively, *p* = 0.54). CO_*CTD*_ and CO_*ITD*_ were closely correlated (*r*^2^ = 0.74), with a mean bias (±SD) of 0.088 ± 0.242 L/min and a percentage error of 19% (Figures 2D, 3D).

## Discussion

Although using the continuous thermodilution method with a Swan-Ganz PAC has been accepted as a gold standard for CO assessment, it has not yet been validated in patients with a continuous-flow LVAD (Haller et al., 1995). In the present study, CO_*CTD*_ was comparable with CO_*ITD*_ while using a PAC in a continuous-flow LVAD in a porcine model of normovolemia, hypervolemia (fluid overload), and hypovolemia (bleeding).

LVAD technology is progressing to improve survival and reduce morbidity in patients with advanced heart failure (Kiamanesh et al., 2019). The specific LVAD strategy may change over time, depending on the patient’s clinical condition, and monitoring CO in these patients will help with decision making.

PAC has been accepted as a gold standard for the clinical measurement of CO (Phillips et al., 2012). It is used predominantly in high-risk surgical and critically ill patients to guide intravenous fluid therapy and inotropic support to improve CO (Kobe et al., 2019). In the last decade, studies have reported excess mortality in patients equipped with a PAC (Shah et al., 2005). However, recently meta-analyses show that, in high-risk surgical patients, the use of a PAC to maintain tissue perfusion decreases mortality and postoperative organ failure (Gurgel and do Nascimento, 2011; Hamilton et al., 2011). Therefore, the PAC is still recommended by the interdisciplinary S3 guidelines on the intensive care of patients following cardiac surgery (Habicher et al., 2018). In the current era, the PAC in patients with LVADs is necessary to establish a specific strategy for each patient, according to their clinical status (Argueta and Paniagua, 2019).

Intermittent thermodilution with PAC has been used as a reference standard for the evaluation of novel CO measurement methods. However, less-invasive CO monitoring devices have percentage errors around 40%, compared to the thermodilution method (Scheeren and Ramsay, 2019). Recently published studies suggest the use of continuous hemodynamic monitoring, especially flow-based variables, such as stroke volume or CO, to prevent occult hypoperfusion and, therefore, decrease morbidity and mortality perioperatively (Bein and Renner, 2019). Continuous thermodilution CO measurement using a modified PAC is being increasingly used. This catheter has a heating filament by which energy is transmitted to the circulating blood (modified thermodilution technique) (Vincent, 2012). This technique has advantages over intermittent bolus thermodilution because intermittent thermodilution CO measurements are affected by variations in injectate volume, rate, and temperature. These variations are eliminated when CO is measured by a continuous thermodilution technique, and it eliminates the need for fluid boluses, reduces contamination risk, requires no operator, and may detect CO change earlier, leading to more rapid decision making (Medin et al., 1998). Several studies support its use in different clinical settings, such as with critical patients, septic shock, cardiogenic shock, bleeding, hypovolemia, hypervolemia, and in the presence of mechanical devices such as the intra-aortic counterpulsation balloon (Lefrant et al., 1995; Medin et al., 1998; Janda et al., 2006). However, it has not yet been validated in patients with a continuous-flow LVAD.

The present study shows that CO_*CTD*_ is comparable with CO_*ITD*_ using PAC during LVAD (bias 315), LVAD and fluid overload (bias 41), and LVAD and acute hemorrhage (bias 88) in a porcine model. This finding is consistent with another study that performed a validation of the CO_*CTD*_ (with PAC) in 15 patients using CO measured through ventricular assist devices (Q-Vue, CCO/SvO2 computer with digital readout) (Mets et al., 2002). There was a bias of 523 mL/min of the CO_*CTD*_ with respect to LVAD flow, therefore, the continuous thermodilution using CAP overestimates CO when compared with LVAD flow. However, this is a variation of approximately 10% of the CO, which is well within the usual range for intermittent bolus thermodilution techniques (Mets et al., 2002). Therefore, results obtained in the study conducted by Mets et al. (2002) are similar to those obtained in the present study. However, they developed their study using a pulsatile-flow LVAD (HeartMate vented electric), and in this study, a continuous-flow LVAD (Biomedicus centrifugal pump) was used. Pulsatile-flow and continuous-flow LVADs show differences in hemodynamic response and ventricular unloading, thus it is necessary to investigate monitoring systems on both LVADs (Cheng et al., 2014).

In the literature we found a study that compares the flow administered by a continuous-flow LVAD (HeartMate II) with the CO obtained by the CAP in patients after implant (day 2–10 postoperative) (Hasin et al., 2014). The CAP overestimated CO by 360 mL/min with respect to the flow of the LVAD with a low correlation (*r* = 0.42). However, LVAD flow estimate (it is generated by a computerized algorithm) may be inaccurate in tracking the true CO, because LVAD flow and native left ventricle flow, both must be correlated with the true CO.

Cardiac output measurement using the CAP in the LVAD can be influenced by atrial or ventricular septal defect, tricuspid regurgitation, or aortic insufficiency (Boerboom et al., 1993; Goldstein et al., 1998). Mini-pigs have a higher incidence of congenital abnormalities (Ho et al., 1991; Quintana-Villamandos et al., 2012), however, in the present study, epicardial echocardiography was performed, and two animals with atrial septal defect were excluded from the study. Epicardial echocardiography showed the absence of tricuspid regurgitation in pigs. This is important because, in severe acute tricuspid regurgitation, the thermodilution method underestimates CO by an amount that is proportional to the level of CO and the grade of regurgitation (Boerboom et al., 1993). The aortic insufficiency can create a circulation loop, where blood is returned directly to the LVAD via the insufficient valve, leading to decreased systemic blood flow and increased left ventricular filling pressure, resulting in falsely high LVAD flow measurements relative to the true systemic flow (Rao et al., 2001). In the present study, epicardial echocardiography also showed the absence of aortic insufficiency in pigs.

In the present study, we have chosen mini-pigs because porcine models are the closest model to human cardiac anatomy, physiology, and specific structures to be encountered during device implantation (pericardium, great vessels, valves, and papillary muscles, blood). They are the gold-standard model to investigate acute left ventricular unloading using a LVAD (Ishikawa et al., 2018), ischemic heart failure by occluding the proximal left anterior descending artery (Ishikawa et al., 2014), pulmonary hypertension by partial pulmonary vein banding (Agüero et al., 2016), left ventricular hypertrophy by banding of the ascending aorta (Ishikawa et al., 2015), or mitral regurgitation by percutaneously severing the mitral valve chordae tendineae (Watanabe et al., 2018).

In conclusion, our study shows that continuous pulmonary thermodilution by PAC could be an alternative method of monitoring CO in a continuous-flow LVAD in a porcine model.

## Limitations and Future Directions

The present study is subject to a series of limitations. First, the LVAD is designed to be used in patients with heart failure; however, we have not induced left ventricle systolic dysfunction because it is possible to study the validity of CO_*CTD*_ for the determination of CO in LVAD in a healthy porcine model (the native ventricle can adjust to LVAD unloading by reducing flow through the aortic valve). The porcine ischemic heart failure model is the most widely accepted heart failure model, however, most studies induce myocardial infarction not sufficient to cause enough dysfunction that develops severe heart failure in clinical situation (patients with LVAD) (Ishikawa et al., 2014). On the other hand, uniform dysfunction is important for the preclinical data interpretation, however, there is inter-animal variability after myocardial infaction (Ishikawa et al., 2014). This could limit the induction of an animal model of ischemic heart failure with uniform dysfunction. In this study, a healthy heart was used, as described elsewhere (Jassawalla et al., 1988; Yamagishi et al., 2001; Kihara et al., 2003; Farrar et al., 2007; Slaughter et al., 2011; Tuzun et al., 2011; Morillas-Sendín et al., 2015), therefore further investigations are necessary to reproduce these results in patients with cardiogenic shock. Second, in thermodilution CO measurements, it is important to note the respiratory cycle, because the CO determined without regard to the respiratory cycle varies 1.4–1.7 L/min. However, the CO measurements were made at end-exhalation when the variations are minimal (Stevens et al., 1985).

## Data Availability Statement

All datasets generated for this study are included in the article/supplementary material, further inquiries can be directed to the corresponding author/s.

## Ethics Statement

The animal study was reviewed and approved by Ethics Committee of Hospital General Universitario Gregorio Marañón, Madrid, Spain.

## Author Contributions

BQ-V and JC conceived the project and wrote the manuscript. BQ-V, MB, IF, MR, and JC performed the experiments, analyzed the data, interpreted the results of experiments, and approved final version of the manuscripts. All authors contributed to the article and approved the submitted version.

## Conflict of Interest

The authors declare that the research was conducted in the absence of any commercial or financial relationships that could be construed as a potential conflict of interest.
